# See to Believe: The Clinical Gaps in the Utility of Ultrasound Imaging in Screening for Ovarian Torsion

**DOI:** 10.7759/cureus.88077

**Published:** 2025-07-16

**Authors:** Joachim Kavalakatt, Jalal Ibrahim, Matthew Thomas, Peter Richa, Hussain Rawiji

**Affiliations:** 1 Oncology, Lake Erie College of Osteopathic Medicine, Bradenton, USA; 2 Dermatology, Lake Erie College of Osteopathic Medicine, Bradenton, USA; 3 Pediatrics and Child Health, Lake Erie College of Osteopathic Medicine, Bradenton, USA; 4 Medicine, Lake Erie College of Osteopathic Medicine, Jacksonville, USA; 5 Pediatrics and Obstetrics, AdventHealth Central Florida, Orange City, USA

**Keywords:** adnexal torsion, clinical decision support, ct imaging, emergency diagnostic laparoscopy, mri imaging, multimodal imaging approach, ovarian torsion, ovarian torsion management, ovary torsion, ultrasound imaging

## Abstract

Ovarian torsion is a gynecologic emergency requiring prompt diagnosis and intervention to prevent ischemic damage and loss of ovarian function. However, its diagnosis remains challenging due to nonspecific symptoms and limitations in imaging modalities. This case report presents two patients with suspected ovarian torsion based on ultrasound findings, both of whom were ultimately found to have alternative diagnoses. One patient underwent surgery, revealing an ovarian endometrioma rather than torsion, while the other was spared unnecessary surgery after MRI findings clarified the diagnosis. These cases underscore the limitations of ultrasound with Doppler as a standalone diagnostic tool and highlight the need for a multimodal imaging approach, incorporating CT or MRI in equivocal cases. A more nuanced analysis is warranted, one that takes into consideration different subpopulations of women by age range and menopausal status to refine clinical management, and also looks at certain diagnostic and imaging features that present within these specific patient groups that can aid in confirming torsion and expediting care. By integrating additional imaging techniques and leveraging emerging diagnostic tools, clinicians can improve accuracy, reduce morbidity, and ensure that surgical intervention is increasingly reserved for true cases of ovarian torsion.

## Introduction

Ovarian torsion is an emergency situation that females can experience, which requires immediate management and treatment. The mechanism of ovarian torsion is when one ovary gets twisted over the ligaments that support the adnexa, specifically the infundibulopelvic (suspensory) ligament, which contains the main ovarian vessels, causing ischemia to occur within the ovary due to a lack of blood supply [1]. Although the ovaries have a dual blood supply from both the ovarian and uterine arteries, the twisting of the ligaments can lead to obstruction of venous outflow initially, then soon after, the arterial inflow will be impacted, leading to swelling, necrosis, infarction, hemorrhage, and potentially peritonitis [1].

The presentation of ovarian torsion can vary from patient to patient depending on age, but most commonly will express unilateral pelvic or lower abdominal pain, which can be sharp, dull, constant, or intermittent [1-2]. For reference, premenopausal patients more commonly present with sharp, stabbing pain near the pelvic area, whereas postmenopausal patients will have dull, constant pain, along with other symptoms such as fever, indicating a necrotic ovary, vomiting, nausea, or abnormal vaginal bleeding [1-2]. With this variety of presentations, it is essential to diagnose ovarian torsion in a timely and effective manner, with correct imaging and labs, then further confirmatory imaging to fully rule it in.

The occurrence of ovarian torsion is relatively low when considering all gynecologic emergencies, with it being only 3% of the emergencies [3]. Interestingly, in a large retrospective analysis of the US emergency department (ED) data that looked at the utilization of the ED for ovarian torsion for one decade, they found the numbers nearly doubled from 1236 patients to 2695 patients [3]. In two separate 10-year reviews, one involving 128 patients and the other 135, only 2.7% of the 128 patients and 15% of the 135 patients who underwent surgery were found to have a clear-cut torsion [4]. Understanding the diagnostic criteria within EDs and determining these low rates of true ovarian torsion within the surgical setting prompts us to dig further to determine a more efficient way to diagnose ovarian torsion without the need to go into the operating room.

In order to efficiently diagnose ovarian torsion, a good history and physical examination should be done first to proceed with further imaging. Although variable, lower abdominal pain/cramping, typically unilateral, is one key element of the history that is worth looking out for. Laboratory testing, such as complete blood count (CBC), comprehensive metabolic panel (CMP), and serum human chorionic gonadotropin (hCG), should always be done to determine the patients’ white blood cell count, hemoglobin (to assess anemia), pregnancy (as this can be a common risk factor for torsion), or other pertinent values within these tests [4]. For imaging guidelines, an ultrasound with Doppler, along with potential transvaginal and pelvic ultrasounds, is done [1-2]. This will give physicians a good idea of the status of the affected ovary, with the sensitivity being around 84% [1]. Typical findings from the imaging will show an enlarged ovary, diminished blood flow, or swelling around the area. Since the ovaries have a dual blood supply, using ultrasound alone does not rule out an ovarian torsion; hence, further imaging with a CT scan or MRI is required to confirm the diagnosis further or to rule out other causes, such as appendicitis [1-2,4]. Ultimately, if a diagnosis is still uncertain due to normal lab values or normal imaging, then a diagnostic laparoscopy, the gold standard, is done [1-2,4].

Management of ovarian torsions is quite simple, with a gynecologist performing surgery to detorse the impacted ovary and evaluate it for its viability; if the ovary is determined to be unsalvageable, then a salpingo-oophorectomy is done [1]. Ovarian torsion can be confused with other differential diagnoses, such as normal pregnancy, ruptured cysts, endometriosis, tubo-ovarian abscesses, or appendicitis [1-2]. It is essential to develop clear-cut symptoms and imaging to rule out an ovarian torsion before performing unnecessary surgery that could be harmful to the patients in the future.

## Case presentation

Patient A

**Figure 1:**
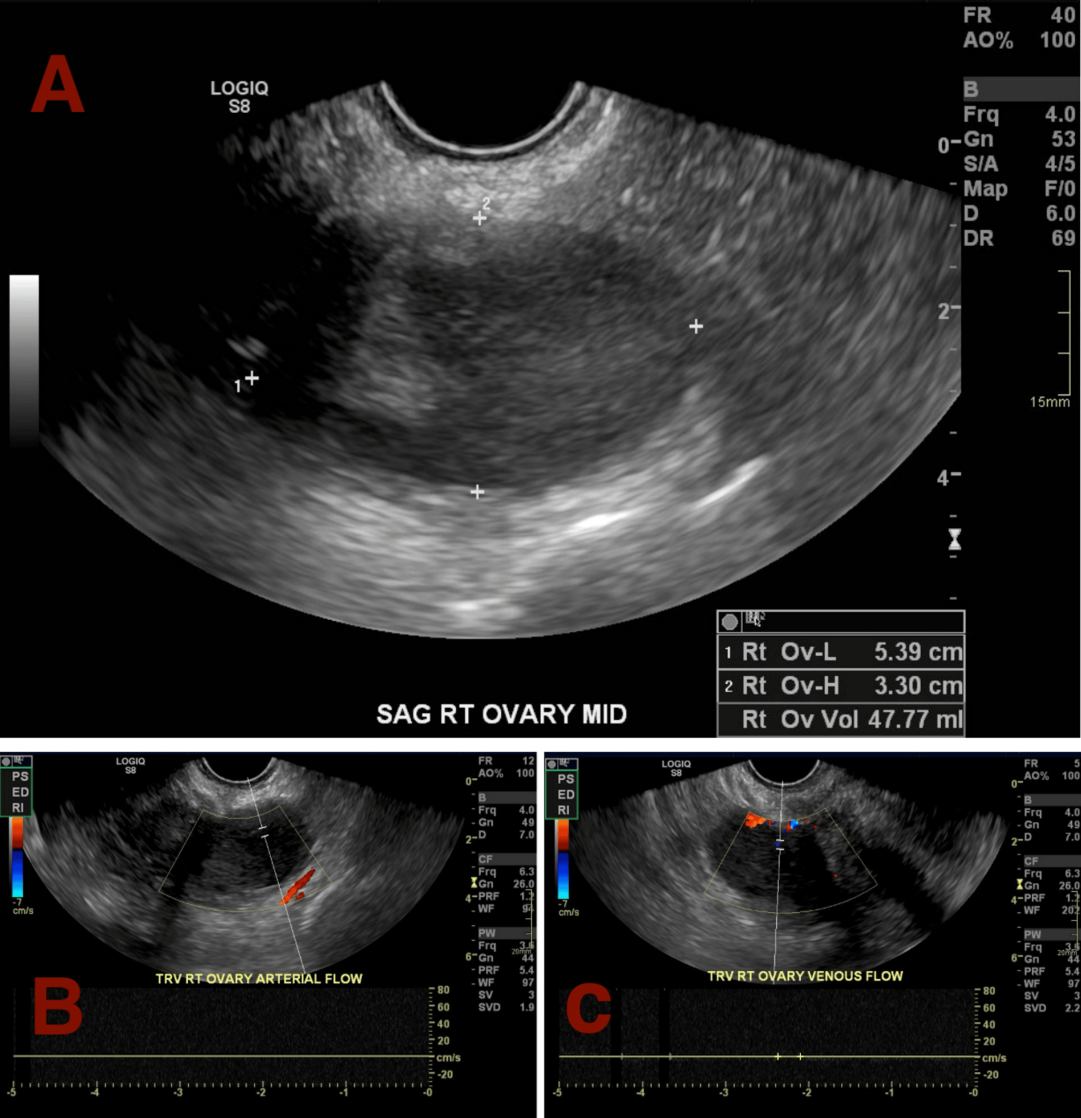
Ultrasound depicting enlarged size for the right ovary (A), measuring at 4.6 × 2.9 × 2.9 cm, followed by Doppler ultrasound depicting loss of arterial (B) and venous (C) flow to the right ovary for Patient A.

**Figure 2:**
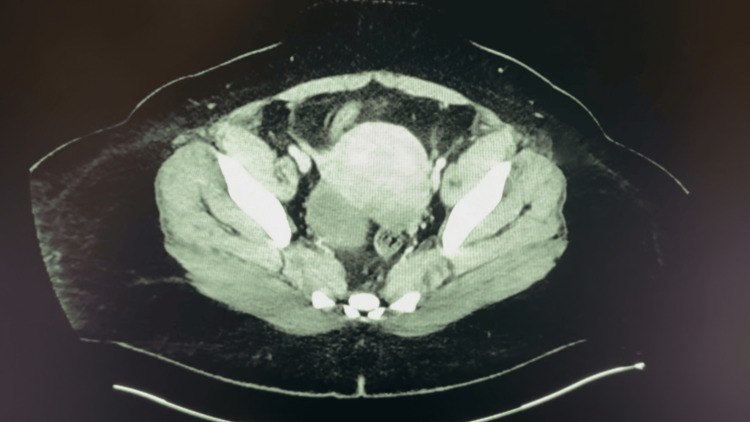
CT imaging of Patient A’s pelvis depicting an enlarged right ovarian cyst measuring 3.1 cm, furthering the diagnosis of ovarian torsion for Patient A.

A 35-year-old nulliparous Hispanic female with a past medical history of anemia, fibroids, obesity, asthma, migraines, kidney stones, and constipation presented to the ED on January 8, 2025, with complaints of intermittent severe left lower abdominal cramping and vaginal bleeding that started last night. The patient's last menstrual period was on December 18, 2024. She also reported passage of vaginal clots. She denied any nausea, vomiting, diarrhea, fever, chills, or urinary symptoms. The patient used Tylenol with minimal relief. Vital signs upon admission showed a blood pressure of 164/96 mmHg, 83 breaths per minute (bpm), a respiratory rate of 18/minute, and a temperature of 98.1°F. Additional lab values expressed a negative pregnancy test, normal WBC, blood in urine, and low mean corpuscular volume (MCV) at 75.6 fL. An ultrasound with Doppler was done, which expressed a slightly enlarged and heterogeneous right ovary measured at 4.6 × 2.9 × 2.9 cm with no arterial or venous Doppler flow (Figure 1). The left ovary was normal in size and had normal blood flow. An additional CT scan of the pelvis also expressed a 3.1 cm right adnexal cyst, which all pointed to the diagnosis of ovarian torsion (Figure 2). This prompted the Obstetrics and Gynecology (OB/GYN) physician to perform a diagnostic laparoscopy to treat and manage the torsion, but upon opening, the physician determined the patient did not present with an ovarian torsion as it was white and not torsed. The physician ended up diagnosing an ovarian endometrioma, which presented as a chocolate-like cyst that ruptured upon surgery. Post-surgery, the physician believed that the imaging may have expressed no blood flow to the cyst, suppressing blood flow to the ovary, making it difficult to determine if it was a true ovarian torsion. Post-operation, the patient is doing well and was discharged on January 11, 2025.

Patient B

**Figure 3:**
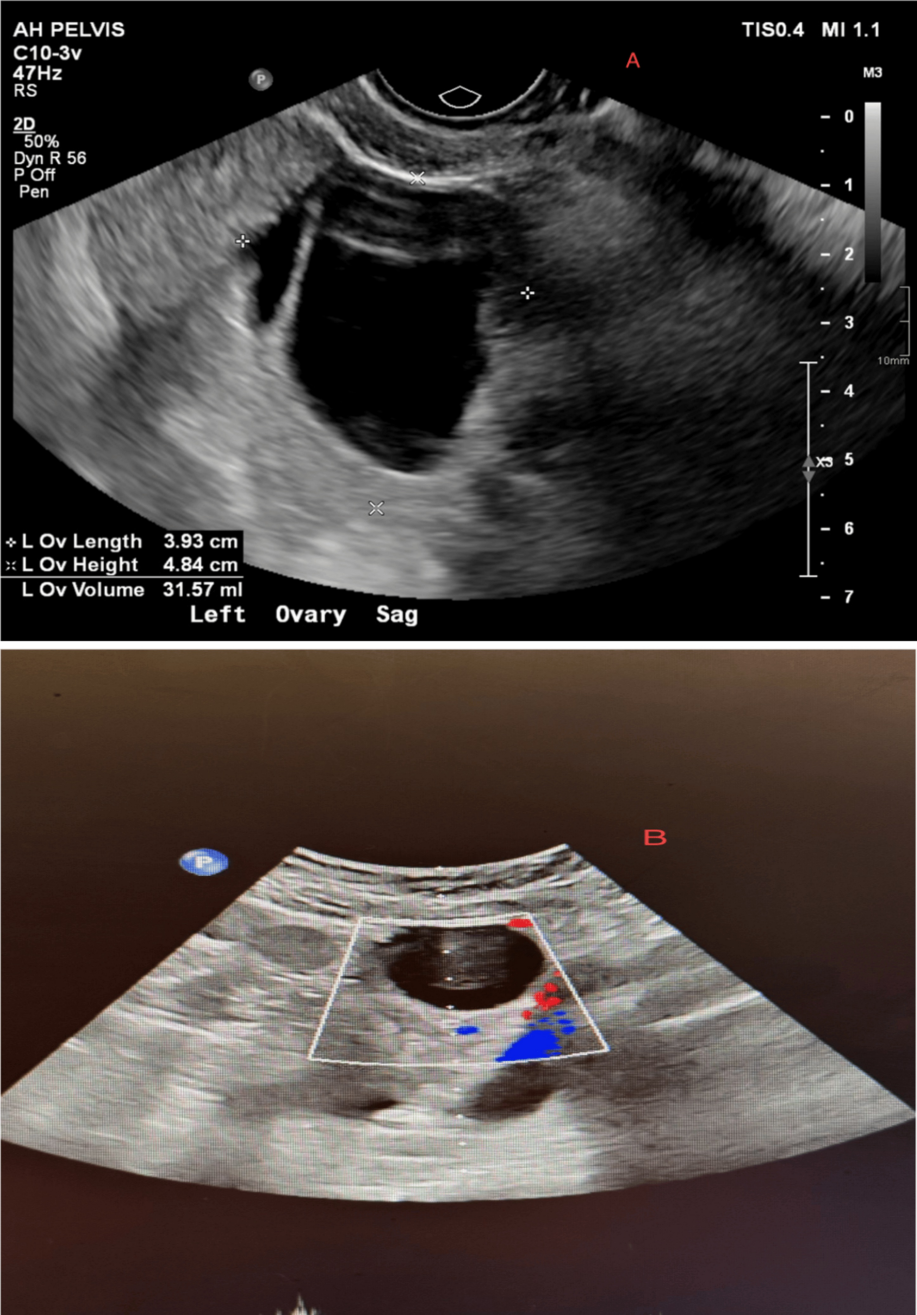
The left ovary was enlarged with a size of 3.8 × 3.2 × 3.9 cm (A). Doppler ultrasound (B) depicting diminished arterial and venous flow to the left ovary for Patient B, due to the potential 4-cm anechoic cyst shown in the upper right of the image.

**Figure 4:**
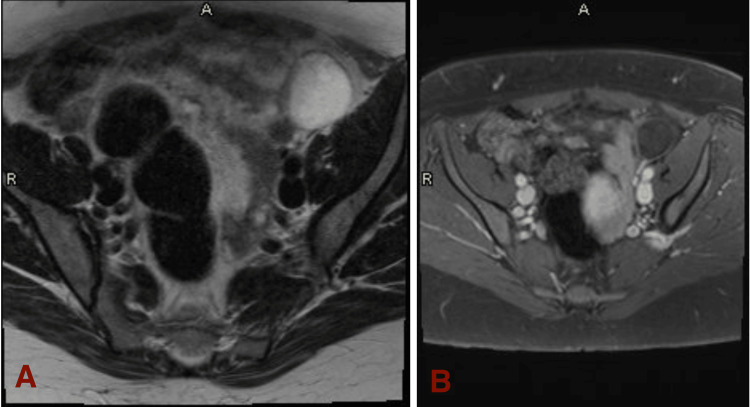
T2-weighted MRI (A) is shown on the left, expressing a simple cyst in the left ovary, measuring about 4 cm in length. The cyst is located in the top right section of the image, white in color. In contrast, the T1-weighted MRI (B) is shown on the right, expressing the same findings but is dark in color. Both MRI images are for Patient B.

A 46-year-old Caucasian female with a past medical history of tuberculosis presented to the ED on January 8, 2025, with complaints of left lower abdominal/pelvic pain with guarding that started one to two hours prior to arrival. She denied any trauma, fever, vomiting, or diarrhea. The patient's last menstrual period was back in November, and she had a previous tubal ligation done. Vital signs upon admission showed a blood pressure of 154/92 mmHg, 91 bpm, and a respiratory rate of 20/minute. Additional lab values showed a negative pregnancy test, low hemoglobin count of 8.7 g/dL, MCV level of 71.8 fL, and a normal WBC. An ultrasound with Doppler was done that showed an enlarged left ovary measuring at 3.8 × 3.2 × 3.9 cm, a 4-cm anechoic cyst in the left ovary, and diminished Doppler flow in the ovary (Figure 3). The ultrasound also expressed a 6.5 × 5.2 × 5.8 cm area of isoechoic to hypoechogenicity in the left adnexa. The OB/GYN physician was consulted but insisted that there be further imaging, such as an MRI, to be done before proceeding with surgery. An MRI was done, which showed no signs of ovarian torsion, but expressed a benign simple cyst in the left ovary measuring about 4 cm, indicating a potential cyst that was eliciting the patient's symptoms and disruption of blood flow on ultrasound (Figure 4). The patient was treated conservatively with hydrocodone-acetaminophen 5-325 mg and Ativan and was soon discharged.

## Discussion

The standard dogma

The road toward an efficient and concise pathway toward the diagnosis of ovarian torsion remains elusive. With a variety of diagnostic modalities and a wide array of differential diagnoses that can complicate patient management, the topic of management of ovarian torsion is anything but simple. We present two cases of suspected ovarian torsion following ultrasound imaging in the adult female population that were subsequently ruled out and excluded as a diagnosis either 1) during the laparoscopic procedure itself or 2) after compelled secondary imaging with MRI. Both of these patient cases were augmented by the use of supplementary modalities after initial ultrasound evaluation, namely, CT imaging (Patient A) and MRI (Patient B). 

According to current guidelines, ultrasound imaging as the first-line modality in the screening of suspected torsion remains the strongest consensus, with highly specific and sensitive visual features (whirlpool sign) displayed that indicate a strong likelihood for ovarian torsion if present [2]. Distinct visual features on ultrasound can lead to a relatively strong suspicion for ovarian torsion and a quick decision to proceed to laparoscopic surgery; several studies have found certain ultrasound signs, such as the whirlpool sign, follicular ring sign, and vascular pedicle twisting, as extremely specific indicators for torsion [2,5]. However, growing evidence also strongly asserts that the lack of these signs on ultrasound imaging, or normal flow on ultrasound imaging with Doppler, cannot allow for the exclusion of ovarian torsion as a diagnosis [2,6]. The relatively lower sensitivities of these features on ultrasound may lead to false positives and an erroneous decision to proceed towards urgent surgery; for example, a retrospective study found that previously held predictive ultrasound signs such as large ovarian volume and ovarian volume ratio still resulted in 43% of patients who were taken into the operative room for suspected torsion that ended up not having true torsion upon visualization through laparoscopy [7]. The acute nature of the presentation of ovarian torsion in the ED setting, in spite of relatively unreliable signs of torsion as seen on ultrasound (such as decreased Doppler flow and vascular edema/congestion), can lead to the decision to pursue surgical management [2,4]. Even after escalation of management to surgery, up to a stunning 50% of laparoscopies find no evidence of torsion, and in some cases, actually lead to significant patient morbidity as a result [2,8]. The recent consensus on management of ovarian torsion points to a more nuanced approach, keeping in mind the patient’s underlying conditions, the use of demographics and subpopulation-based outcomes, and the use of multiple imaging techniques to provide for higher sensitivity and specificity in guiding management for suspected torsion.

Differential diagnoses

The presence of confounding gynecological pathologies, from uterine fibroids to dermoid cysts, can have dramatic effects on the likelihood of ovarian torsion. A detailed clinical history alongside a careful evaluation of previous underlying conditions must be taken into account before proceeding to laparoscopy. Polycystic ovarian syndrome (PCOS) and premenopausal status are some of the most common risk factors for ovarian torsion, and likewise, patients with these conditions are at increased risk of premature surgical management and its subsequent morbidity and complication risks, and thus warrant a more diligent screening regimen for ruling out torsion when suspected [9]. Similarly, ovarian cysts (as seen in Patient B) and other pathologies promoting bulk-related symptoms can easily propagate torsion and serve as a lead point for torsional stress on the infundibulopelvic or utero-ovarian ligaments [6]. Endometriosis (as seen in Patient A) can also promote conditions leading towards torsion, with progressive growth of adhesions and endometrioid cysts (endometriomas), which significantly increase torsion risk in a similar manner [10]. As stated previously, a thorough clinical history is key towards isolating ovarian torsion as the most likely diagnosis, yet even after this step, torsion cannot be absolutely confirmed as a diagnosis. 

Patient subpopulations in guiding management

An evaluation of the current guidelines in the management of ovarian torsion points to an inefficiency in the clinical assessment of a patient’s likelihood of presenting with a true ovarian torsion. The diversity of patient cases that present with torsion is not to be ignored, and a “one size fits all” approach in managing different patients simply does not suffice. For example, whether presenting patients are premenopausal or postmenopausal has a significant degree of impact on management. Several studies have found that premenopausal patients and younger female patients display longer intervals from symptomatic onset to hospital admission, exhibit increased blood loss from surgery, and are observed to have longer hospital stays compared to postmenopausal patients for suspected ovarian torsion [11]. Thus, these patients may require more diligent screening before proceeding with further management [11]. Similarly, previously mentioned specific signs on ultrasound can work particularly well for certain subpopulations of patients; for example, ovarian volume ratio (previously mentioned to not be as effective in preventing adolescent patients without true torsion from proceeding to surgery) and peripheralized follicles were found to be uniquely and overwhelmingly specific in detecting true ovarian torsion in premenarchal female and pediatric patients [7,12]. The increased weighted importance of certain imaging signs based on the presenting patient’s demographics and subpopulation status can allow clinicians to make much more reliable decisions in their approach to managing torsion. 

Optimization of multiple imaging techniques

Likewise, the reliance on multiple imaging modalities allows physicians to more accurately gauge the likelihood of a true torsion and the necessity for surgical intervention, and in the midst of the variable presentations of ovarian torsion, a combination approach is increasingly warranted. The use of MRI and CT scans as supplementary and confirmatory imaging for suspected ovarian torsion has been established, but several studies have recently pointed to various MRI protocols, MRI-based scoring systems, and predetermined imaging sequence systems that can be used to predict ovary necrosis and diagnose torsion rapidly and more efficiently than when compared to physician interpretation alone [13,14]. Recent trials support the use of rapid MRI as a suitable candidate for supplemental imaging for suspected torsion in an acute emergency setting [15]. Remarkably, the development of promising machine learning models and language processing models that can indicate the likelihood of detecting torsion based on patient demographics, risk factors, ultrasound and CT/MRI image data, and more has been shown to further enhance management (improving true detection of torsion to 84% from 74% in one study) and potentially decrease the risk of unnecessary surgery, mortality, and morbidity (although further studies are needed in this domain) [16,17]. While there is increasing evidence supporting the use of MRI and CT scans, composite scoring systems, sequential screening algorithms for torsion, and artificial intelligence, these options do not come without limitations. Clinicians must carefully navigate the risks of increased radiation to the patient from employing additional scans (especially in pediatric/adolescent patients) and the further time needed to perform these scans, obtain scores, and run analyses in the setting of an acute presenting emergency [16]. 

Other considerations

While ovarian torsion is symptomatically and clinically considered an acute diagnosis and dangerous if not emergently managed, a few recent studies challenge this notion. One retrospective study found no significant difference in physical appearance, examination, ultrasound findings, and rate of adnexal ischemia between cases of torsion managed by immediate (within six hours of symptom onset) and late (from six to 24 hours after symptom onset) surgical intervention [18]. Another recent retrospective study revealed no significant association between the time elapsed between the onset of symptoms up until the start of surgery and the degree of ovarian loss from torsion; in other words, the time taken to rush patients into surgery from the moment of suspecting ovarian torsion in the ED (early times being within an hour of since diagnosis and late being after an hour since diagnosis) did not significantly affect the degree of ovary loss [19]. Building on this consensus, a recent study found that histopathological grading of 31 excised ovaries from suspected cases of ovarian torsion revealed that only five of these ovaries exhibited true necrosis [20]. These findings, while limited by their retrospective nature, may indicate that despite the symptomatic urgency of torsion, there may be room to take some more time for a more thorough clinical evaluation before the decision to pursue surgery is made.

## Conclusions

The cases of Patient A and Patient B highlight an important challenge in diagnosing ovarian torsion. Ultrasound with Doppler, while a valuable first-line tool, is not always definitive. Patient A underwent surgery based on ultrasound findings, only to reveal an ovarian endometrioma rather than torsion, whereas Patient B was spared an unnecessary operation due to further imaging with MRI. These cases serve as a reminder that while ultrasound can strongly suggest torsion, it is not infallible, and its limitations can lead to misdiagnosis and unnecessary surgical interventions. In an emergency setting, the urgency to act quickly is understandable, but a more thoughtful approach is needed. Instead of rushing to surgery based on ultrasound findings alone, incorporating additional imaging such as CT or MRI in equivocal cases can improve diagnostic accuracy and prevent avoidable procedures. This multi-modal approach allows physicians to distinguish true torsion from other gynecologic conditions that mimic its presentation, ultimately leading to better patient outcomes. Moving forward, we need to refine our diagnostic protocols to ensure that surgical intervention is reserved for those who truly need it. By integrating secondary imaging and leveraging advancements in artificial intelligence and multimodal technology, we can create a more efficient and accurate pathway for diagnosing ovarian torsion. The goal is not just to act quickly, but to act wisely, balancing the need for timely treatment with the responsibility of avoiding unnecessary harm to our patients.

## References

[1] Baron SL, Mathai JK (2023). Ovarian torsion. StatPearls.

[2] ACOG Committee (2019). Adnexal torsion in adolescents: ACOG Committee opinion no, 783. Obstet Gynecol.

[3] Tabbara F, Hariri M, Hitti E (2024). Ovarian torsion: a retrospective case series at a tertiary care center emergency department. PLoS One.

[4] Huang C, Hong MK, Ding DC (2017). A review of ovary torsion. Tzu Chi Med J.

[5] Yatsenko O, Vlachou PA, Glanc P (2021). Predictive value of single or combined ultrasound signs in the diagnosis of ovarian torsion. J Ultrasound Med.

[6] Bridwell RE, Koyfman A, Long B (2022). High risk and low prevalence diseases: ovarian torsion. Am J Emerg Med.

[7] Roberts B, Golden J, Kallis M, Denning NL, Lipskar AM, Rich BS (2022). Operative findings in pediatric and adolescent patients with presumed adnexal torsion. J Surg Res.

[8] Meljen V, Fridman D (2020). Gynecologist’s perspective: semantics of “ruling out” ovarian torsion. J Ultrasound Med.

[9] Psilopatis I, Damaskos C, Garmpis N (2023). Ovarian torsion in polycystic ovary syndrome: a potential threat?. Biomedicines.

[10] Hua D, Zhao P, Jiang L (2019). Torsion of ovarian endometrioma in pregnancy: a case report and review of the literature. Trop Doct.

[11] Li M, Wang H, Hou S, Wang S, Li H (2024). Comparison of characteristics and outcomes of premenopausal and postmenopausal women with adnexal torsion. J Int Med Res.

[12] George JS, Rosen MW, Curci N, Torres ML, Wasnik AP, Smith YR, Quint EH (2023). Sonographic predictors of ovarian torsion in premenarchal girls. J Pediatr Adolesc Gynecol.

[13] Wattar B, Rimmer M, Rogozinska E, Macmillian M, Khan KS, Al Wattar BH (2021). Accuracy of imaging modalities for adnexal torsion: a systematic review and meta-analysis. BJOG.

[14] Renganathan R, Subramaniam P, Deebika S, Arunachalam VK, Shanmugam J, Cherian M (2023). Scoring system for predicting ovarian necrosis in adnexal torsion using an ultra-short optimized MRI protocol. Abdom Radiol (NY).

[15] Epstein KN, Trout AT, Debnath P (2024). Rapid, free-breathing non-contrast MRI for first-line imaging evaluation of ovarian torsion in the emergency department. Pediatr Radiol.

[16] Yagur Y, Brisker K, Kveler K (2023). Can natural language processing improve adnexal torsion predictions?. J Minim Invasive Gynecol.

[17] Otjen JP, Stanescu AL, Alessio AM, Parisi MT (2020). Ovarian torsion: developing a machine-learned algorithm for diagnosis. Pediatr Radiol.

[18] Yaakov O, Ashwal E, Gemer O (2022). Acute adnexal torsion: is immediate surgical intervention associated with a better outcome?. Gynecol Obstet Invest.

[19] Avila A, Motta M, Schechter D (2024). Ovarian salvage with prompt surgical intervention for adnexal torsion: does timing matter?. Am Surg.

[20] Novoa M, Friedman J, Mayrink M (2021). Ovarian torsion: can we save the ovary?. Arch Gynecol Obstet.

